# Menschenwürde und Autonomie bei medizinethischen Entscheidungen am Lebensende

**DOI:** 10.1007/s00391-024-02308-1

**Published:** 2024-05-14

**Authors:** Florian Derler

**Affiliations:** Klinik für Palliativmedizin, Universitätsklinikum Heidelberg, Im Neuenheimer Feld 305, 69120 Heidelberg, Deutschland

**Keywords:** Würdezentrierte Patientenversorgung, Mutmaßlicher Patientenwille, Selbstbestimmung, Sterbebegleitung, Klinische Ethik, Dignity-centered patient care, Surrogate decision-making, Self-determination, Terminal care, Clinical ethics

## Abstract

Das Respektieren der Menschenwürde ist zweifellos ein zentraler Bestandteil des klinischen Alltags und von medizinethischen Evaluationen. Manche Aspekte der Menschenwürde unterliegen im Laufe des Lebens einer gewissen Wandlung. Wenn die Fähigkeit zur freien Willensbildung eingeschränkt ist, kann es erschwert sein, den individuellen Vorstellungen einer würdevollen Behandlung gerecht zu werden, was speziell am Lebensende an Bedeutung gewinnt. Anhand einer Kasuistik soll demonstriert werden, welche moralischen Problemstellungen auftreten können, wenn aktuelle Willensbekundungen des Patienten gegensätzlich sind zu dem, was vorausverfügt wurde oder den Angehörigen vertraut ist. Bei der klinischen Entscheidungsfindung kann die Betonung der Willensfestlegung in einer Patientenverfügung so weit führen, dass Menschenwürde auf Autonomie reduziert wird. Ein auf Autonomie reduziertes Konzept der Menschenwürde geht aber am Kern sowohl einer würdezentrierten Medizin als auch der partizipativen Entscheidungsfindung vorbei. Komplementäre Aspekte der Menschenwürde, wie beispielsweise Authentizität und Selbstaktualisierung, müssen berücksichtigt werden. Außerdem sollte die Medizin als solche den Tod nicht marginalisieren, sondern eine Versorgung, die die Prinzipien der menschlichen Interaktion und einer umfänglichen Auffassung von Würde fokussiert, anbieten.

**Zusatzmaterial online:**

Zusätzliche Informationen sind in der Online-Version dieses Artikels (10.1007/s00391-024-02308-1) enthalten.

## Abstract (en)

Respect of human dignity is an unquestionable part of daily clinical routine as well as of evaluations in medical ethics. Some aspects of human dignity are a subject of transformation in the course of a lifetime. If the ability to form a will is reduced, it might be difficult to satisfy the individual perception of a dignified treatment, which is of particular interest in terminal care. As illustrated by the presented case report, moral problems can arise when the patient’s present verbal or non-verbal expressions are opposed to what is documented or what is estimated by dependants to be the individual’s will. In clinical decision-making processes, the determination of the will via an advance directive can be overstated, which can go so far that human dignity is reduced to autonomy. A concept of dignity reduced to autonomy misses the point not only of a dignity-centered medical care but also of shared decision-making. In situations at the end of life other aspects, such as authenticity or self-actualization should be considered when respecting the individual’s dignity. Medicine should as such not marginalize death but provide a care based on principles that focus on human interaction and respect for a comprehensive concept of dignity.

## Einleitung

Zur Umsetzung des 25. Artikels der UN-Menschenrechtsdeklaration (Recht auf Gesundheit) wurde der International Covenant on Economic, Social and Cultural Rights (ICESCR, kurz: Sozialpakt) völkerrechtlich verpflichtend eingesetzt. Der dort zu findende 14. General Comment präzisiert, dass das Recht auf den höchsten erreichbaren Gesundheitsstandard Folgendes beinhaltet: „attention and care for chronically and terminally ill persons, sparing them avoidable pain and enabling them to die with dignity“, also die Zuwendung und Pflege für chronisch und terminal kranke Personen, indem ihnen Schmerz erspart und ein Sterben in Würde ermöglicht wird [13]. Aus dem Menschenrecht auf Gesundheit werden folglich auch ein Recht auf ein würdevolles Sterben und ein normativer Anspruch abgeleitet.

Was es für ein *würdevolles Sterben* braucht, ist Zentrum einer Debatte im medizinethischen Diskurs zwischen den Prinzipien der Menschenwürde und der Autonomie, wie sie insbesondere im angloamerikanischen Sprachraum zu finden ist. In diesem Artikel wird das Spannungsfeld zwischen Menschwürde und Autonomie auf die deutsche Situation im Kontext aktueller medizinethischer bzw. -rechtlicher Diskussionen eingeordnet und der Status quo analysiert.

Mit Fokus auf die ethischen Aspekte werden in diesem Artikel die Vielschichtigkeit und Ambiguität der Konzepte der Menschenwürde und Autonomie erläutert, um eine differenzierte Sichtweise auf die Debatte anzuregen und die Anwendung auf konkrete moralische Konfliktsituationen im klinischen Alltag zu erleichtern. Dabei geht es nicht darum, eine Beurteilung in Bezug auf *richtig* oder *falsch* in der ethischen Entscheidungsfindung zu erlauben, sondern eine persönliche Analyse und Reflexion der zur Debatte stehenden Prinzipien im konkreten Fall zu ermöglichen, um so ethische Erwägungskompetenz in vergleichbaren klinischen Situationen zu fördern.

### Aspekte der Autonomie am Lebensende

Autonomie ist jene Fähigkeit eines Akteurs^1^, seinen Willen kraft der Vernunft selbst und frei zu bestimmen, um so zum Erhalt der Würde beizutragen (ausführlichere Überlegungen finden sich z. B. bei [2, 6]). Wo aber der Mensch an der Ausübung elementarer Formen der Selbstbestimmung gehindert wird, z. B. aufgrund von Zwang oder krankheitsbedingter Unfähigkeit, ist die Menschenwürde eingeschränkt und bedroht. Warum der Selbstbestimmungsfähigkeit besonders in der eher liberal-rechteorientierten Medizinethik ein oftmals prioritärer Rang beigemessen zu werden scheint (Stichwort: Partikularmoral), kann damit begründet werden, dass sie das einzige unter den vier medizinethischen Prinzipien ist, das primär vom Patienten selbst mit seinen individuellen Wertvorstellungen ausgeht und welches gegen den negativ konnotierten ärztlichen Paternalismus wirkt [1].

Autonomie als Selbstbestimmung ist also eine entscheidende Komponente der Menschenwürde. Dies kann sogar so weit gehen, dass hinter der Menschenwürde ein nutzloses Konzept, welches nichts anderes als die Achtung der Autonomie bedeutet, vermutet wird [16, 21]. Bezogen auf das Lebensende wird in der Tat schwindende Selbstbestimmungsfähigkeit (hier im Sinne von abhandenkommender Kontrolle) von Patienten oftmals als erheblich würdebeeinträchtigend erlebt und der Aufrechterhaltung von Autonomie eine große subjektive Wertschätzung beigemessen [19, 20]. In der Debatte zur Suizidassistenz wird auch im deutschsprachigen Raum auf der Basis desselben Prinzips der Würde argumentiert, dass einerseits erst kraft der Autonomie ein würdevolles Sterben erwirkt werden kann, andererseits die Respektierung der Würde dem selbstbestimmten Recht auf Selbsttötung zuwiderläuft [7, 22, 25].

Ob dieser hier skizzierte Reduktionismus der Menschenwürde auf Autonomie der Komplexität der Sache gerecht wird, soll im Folgenden behandelt werden. Außerdem wird aufgezeigt, warum es sich lohnt, einen differenzierteren Blick auf die Menschenwürde (am Lebensende) und den Zusammenhang zur Selbstbestimmung zu wagen, und welche zugrunde liegenden Überlegungen eine Rolle spielen, wenn autonom verstandene Akte im Widerspruch zu gängigen Konzepten von Menschenwürde stehen.

### Autonomie vs. Menschenwürde?

Es gibt Hinweise, dass gerade in Bezug auf Tod und Sterben die Autonomie aus Patientenperspektive gar nicht so prioritär eingeschätzt wird, wie auf der Grundlage der prinzipienorientierten Ethik vermutet, sondern andere Themen wie eben die Würde oder die Art der sozialen Interaktion mit Familie, Behandlern etc. als vordergründig erachtet werden [18, 23]. Autonomie und Würde werden so wie im Leben auch beim Sterben mitbestimmt von sozialen Eventualitäten, wie sie im *Zusatzmaterial online: Supplement 1* weiter erläutert und in den Kontext der spezifischen Würde am Lebensende eingebettet werden. Dabei spielen Überlegungen zur *Authentizität* und *Integrität* eine wesentliche Rolle. Nicht zuletzt kann kraft der Autonomie bereits im Voraus ein wesentlicher Beitrag zum Schutz der individuellen Auffassung von Menschenwürde in Form einer Vorsorgevollmacht oder Patientenverfügung geleistet werden.

Die folgende fiktive Kasuistik soll dazu dienen, die vorangestellten Überlegungen und die nachfolgende Diskussion in einen praxisnahen, medizinethisch relevanten Kontext zu setzen.

## Kasuistik

Ein 84-jähriger Patient erlitt einen Schlaganfall und wies als Folge eine leichte Halbseitenlähmung rechts mit Rollatormobilität und eine Aphasie mit erhaltenem Sprachverständnis auf. Außerdem war noch eine Dysphagie, welche bereits vor dem Schlaganfall in leichter Ausprägung bestanden haben soll, festzustellen. Während der Rehabilitation kam es zu einer Lungenentzündung auf dem Boden der weiterhin ausgeprägten Schluckstörung. Die Pneumonie wurde erfolgreich behandelt, doch wurde aufgrund der Schluckstörung die Indikation zur Anlage einer Magensonde gestellt. Für Unsicherheit sorgte, dass in der Patientenverfügung lebensverlängernde Maßnahmen wie eine Magensonde abgelehnt wurden. Durch Kommunikation mittels Kopfnicken und -schütteln lehnte der Patient eine Magensonde ab. Da zwischenzeitlich ein delirantes Syndrom bestand, schätzte das Behandlungsteam den Patienten als nichteinwilligungsfähig ein. Man entschloss sich im Konsens mit den Angehörigen zur Anlage einer nasogastralen Magensonde mit der Begründung, dass die Patientenverfügung in der vorliegenden Situation aufgrund des temporären Charakters bis zum Wiedererlangen einer suffizienten Schluckfunktion nicht greife. Der weiterhin delirante Patient zog wiederholt die Magensonde, beim erneuten Legen entstanden kleinere Schleimhautverletzungen, was schließlich freiheitsentziehende Maßnahmen in Form des Tragens von „Delirhandschuhen“ nach sich zog.

Während der Anlage einer perkutanen enteralen Gastrostomie (PEG), als aufgrund einer akuten Anämie eine Magenspiegelung erforderlich wurde, entdeckte man einen malignen Tumor der Speiseröhre im nicht mehr kurativen Stadium. In den folgenden Tagen stabilisierten sich sowohl der körperliche Zustand als auch die durch Schlaganfall und Tumor bedingte Schluckstörung des Betroffenen deutlich, und das Delir klang ab. Die Angehörigen plädierten nun für die Entfernung der PEG-Sonde mit dem Argument, dass die Voraussetzung einer unheilbaren Erkrankung gemäß Patientenverfügung gegeben sei und der Patient wieder über eine ausreichende geistige Verfassung verfüge. Auch das Behandlungsteam war dieser Ansicht. Der 84-Jährige selbst konnte seinen Willen weiterhin nonverbal kundtun und lehnte eine palliative Chemotherapie ab. Entgegen dem in der Patientenverfügung dokumentierten Willen zeigte er aber nicht nur eine Akzeptanz gegenüber der PEG-Sonde, sondern erlernte sogar bereitwillig deren Bedienung.

Da Einwände gegen die Verwertbarkeit der Willensäußerungen des Patienten gemacht wurden und auch den Angehörigen die Diskrepanz zwischen Verweigerung der Chemotherapie und Akzeptanz der PEG-Sonde nicht nachvollziehbar erschien, kam es zur klinischen Ethikberatung. Im Gespräch mit den Angehörigen kam zum Vorschein, dass der Mann immer schon sehr auf Selbstständigkeit bedacht gewesen sei und sich auch im Alter noch gern mit technischen Neuerungen auseinandergesetzt habe. Außerdem sei er ein gläubiger Mensch, der nach dem Tod seiner Ehefrau eine gelassene Haltung zum Thema Sterben entwickelt habe. Ein psychiatrisches Konsil beurteilte die Einwilligungsfähigkeit des Patienten als eingeschränkt, aber nicht aufgehoben, da er das Für und Wider der PEG-Sonden-Beibehaltung erfassen und abwägen könne, sich aber potenziell daraus resultierender Komplikationen nicht sicher gewahr sei. Man kam zu der Übereinkunft, dass der Patientenautonomie der Vorrang gegenüber der Fürsorge (palliative Chemotherapie) und dem Nichtschadensprinzip (Komplikationsrisiko der PEG-Sonde bei Selbstbedienung) gegeben werden sollte. Unter palliativen Maßgaben und mit einliegender PEG-Sonde wurde der Patient schließlich in ein Hospiz verlegt und entwickelte einen Monat später eine PEG-Sonden-assoziierte Bauchdeckenentzündung mit letalem Ausgang.

## Diskussion

Anhand des Fallbeispiels wurde auf die Probleme eines oft zu reduktionistisch gedachten Konzepts von Autonomie und anderen ethischen Prinzipien aufmerksam gemacht. Reduktionistisch meint hier eine ausschließlich auf Vernunft und freier Willensbildung basierende Autonomie, die andere Teilaspekte wie die Würdigung der Biografie oder der Authentizität bei der Gesamtbeurteilung der Menschenwürde missachtet.

Die hier folgende Diskussion soll analysieren, ob ein Konflikt bzw. eine Redundanz zwischen den beiden Konzepten Menschenwürde und Autonomie überhaupt auf den deutschen Diskurs übertragbar ist. Ausgehend von der Kasuistik möchte der Autor zudem einen Anstoß für eine kritische Reflexion sowohl der zur Debatte stehenden ethischen Prinzipien als auch der Standpunkte der involvierten Akteure geben (*Zusatzmaterial online: Supplement 1*).

### Selbstbestimmung und Menschenwürde anhand medizinrechtlicher Aspekte zu Zwangsmaßnahmen und selbstbestimmtem Sterben

Während sich im angloamerikanischen Raum die ethische Debatte teilweise zuspitzt auf einen Konflikt zwischen Autonomie und Menschenwürde aufgrund postulierter Redundanz der beiden Konzepte, scheint die Diskussion in Deutschland differenzierter zu sein. Die Relevanz der Thematik für den klinisch-ethischen wie auch rechtlichen Diskurs ist hochaktuell, gestaltet sich aber eher innerthematisch konfliktbeladen, wie folgend gezeigt werden soll.

Rechtsbezogen sind die §§ 1827 –1829 des Bürgerlichen Gesetzbuches (BGB) in dem präsentierten klinischen Fall maßgeblich. Neben der Ermittlung des mutmaßlichen Patientenwillens anhand konkreter Anhaltspunkte (§ 1827 Absatz 2 Satz 1 BGB), betont der darauffolgende § 1828 Absatz 1 und 2 BGB den partizipativen Charakter der Entscheidungsfindung. Prominent zum Vorschein kommt dabei die Betonung der Autonomie, die moralische, religiöse und andere Wertvorstellungen des Betreuten miteinbezieht. Ohne explizite Nennung der Würde erlaubt diese Erläuterung doch zumindest einen Rückschluss auf eine erweiterte Perspektive der Autonomie, die individuelle Würdeaspekte des Betroffenen anerkennt. Ein reduktionistisches Verständnis der Menschenwürde findet man hier nicht, wenngleich die Autonomie eine prominente Stellung einnimmt.

Wie im Fallbeispiel handelt es sich bei Entscheidungen am Lebensende zumeist um Menschen, die von bestimmten Behinderungen betroffen sind. Rückt man dabei die Selbstbestimmungsfähigkeit dieses Personenkreises in den Fokus, ist zu berücksichtigen: Der eingangs erwähnte Sozialpakt hat in Deutschland aufgrund seines Status als ratifizierter Menschenrechtsvertrag Rechtswirkung. Das Institut für Menschenrechte ist mit dem Monitoring der Umsetzung der UN-Behindertenrechtskonvention (UN-BRK) betraut, die UN Open-Ended Working Group on Ageing (OEWG-A) soll dem besonders berücksichtigungswürdigen Schutz vulnerabler Personengruppen im Kontext von Entscheidungen am Lebensende Rechnung tragen. Dabei ist insbesondere der Artikel 25 der UN-BRK von Relevanz. Demgemäß findet sich im Zuge der Diskussion Autonomie und Selbstbestimmung an der Spitze der Prinzipien die Menschenwürde. Explizit wird dabei auch die Möglichkeit der persönlichen Zustimmung zur Gestaltung des eigenen Lebensendes erwähnt, die zwar durch Unterstützung gewährleistet, aber, wenn möglich, nicht durch Zustimmung anderer ersetzt werden soll [10]. Betont wird in diesem Zusammenhang auch die selbstbestimmte Lebensführung, die ohne fördernde und unterstützende soziale Strukturen nie gelingen kann. In den Artikeln 9 und 12 tritt das Autonomieverständnis der UN-Menschenrechtskonvention als Prinzip zum Vorschein [11, 12]: Ohne explizite Angabe des Wortes Autonomie lässt sich erkennen, dass die Autonomie untrennbar mit der Menschenwürde und dem Gleichheitssatz verwoben ist. Erneut ist zwar keine Reduktion auf, aber doch eine dominierende Rolle der Autonomie in der Auffassung der Menschenwürde zu finden.

Gerade in der Umsetzung der Vorgaben des Artikels 12 der UN-BRK kam es mit dem zum 01.01.2023 wirksamen Gesetz zur Reform des Vormundschafts- und Betreuungsrechts zu einer Stärkung des Selbstbestimmungsrechts in Deutschland. Ziel ist es, die Selbstbestimmung von betreuten Menschen und die Qualität der rechtlichen Betreuung zu stärken. Wie in der dargestellten Kasuistik kann es in der klinischen Praxis in der Wahrnehmung der aus Artikel 2 Absatz 2 Satz 1 des Grundgesetzes folgenden Schutzpflicht des Staates zu einem Konflikt zwischen Schutzpflicht und auf Autonomie beruhendem Selbstbestimmungsrecht kommen, wenn der natürliche Wille einer nichteinsichtsfähigen Person konträr zu einer medizinisch indizierten Behandlung steht [4]. Neben dem aufklärenden und möglichst empathischen Arztgespräch kann v. a. auch die klinische Ethikberatung dazu beitragen, den Konflikt zwischen den kollidierenden Freiheits- und Schutzprinzipien des Grundrechts desselben Rechtsgutträgers aufzulösen.

Entscheidungen am Lebensende sind im Zuge des Bundesverfassungsgerichtsurteils vom 26.02.2020, im Rahmen dessen der § 217 des Strafgesetzbuchs (StGB) zum Verbot der Suizidhilfe für nichtig erklärt wurde, in den Fokus des öffentlichen Interesses gerückt [5]. Im vergangenen Jahr erhöhte die Debatte mit den letztlich gescheiterten Gesetzesvorschlägen im Bundestag die Relevanz des medizinethischen und auch gesellschaftspolitischen Diskurses. Bei der Verwirklichung des *Sterben-in-Würde*-Postulats wird unter Verweis auf das Prinzip *Würde* von den einen der Selbstbestimmung des Einzelnen eine höhere Priorität gegenüber dem Lebensschutz eingeräumt, während es sich für die Opposition genau umgekehrt darstellt [3, 27].

Auch wenn das Bundesverfassungsgericht das Autonomieprinzip als maßgeblich für die juristische Bewertung erachtete, bezog sich das Urteil explizit auf das Prinzip der Menschenwürde, zu welcher auch das Recht gehöre, freiverantwortlich und unter Zuhilfenahme Dritter seinem Leben ein Ende zu setzen. Ganz konkret heißt es dort: „Der grundgesetzlich geschützte Gehalt der Menschenwürde dürfe ferner deshalb nicht auf absolute Autonomie des Einzelnen verkürzt werden, weil die Menschenwürde gerade auch Menschen zukomme, die nicht (mehr) zur Selbstbestimmung fähig seien“ [5]. Neben objektiven Ansprüchen der Menschenwürde kommen auch individuelle Aspekte sowie die der Menschenwürde innewohnenden Idee autonomer Persönlichkeitsentfaltung nicht zu kurz (hierzu Rn. 210 und 211 [5]). Es lässt sich feststellen, dass dem höchstrichterlichen Urteil kein reduktionistisches Konzept der Menschenwürde zugrunde gelegt wurde und es eben die vielschichtigen Aspekte der Menschenwürde sind, denen die Rechtssache gerecht werden soll. Dennoch fehlt nach dem Scheitern der Gesetzesentwürfe im Bundestag bis dato ein juristisch verbindlicher Rahmen, der das Recht auf selbstbestimmtes Sterben regelt und Betroffene, Mediziner und alle übrigen Involvierten nicht mehr im Stich lässt. Daher ist, übertragen aus dem Fallbeispiel und der Debatte um das selbstbestimmte Sterben, abschließend klarzustellen, dass gegenwärtig gerade aufgrund der ungeklärten Rechtslage juristischer Rat einzuholen wäre, wenn man in der Praxis vor einer vergleichbaren Konfliktsituation steht.

### Ethische Gedanken zu Autonomie und Menschenwürde am Lebensende

Unter anderem aufgrund des technischen und wissenschaftlichen Fortschritts der Medizin im 20. Jh. konnten auch bei sonst sicher tödlich verlaufenden Erkrankungen die Prognose verbessert und das durchschnittliche Lebensalter angehoben werden [9]. Das Sterben wird kaum noch als natürlicher Lebensabschnitt wahrgenommen. Für die meisten Menschen führte die Vorstellung eines Sterbens angesichts der apparativen Dominanz in Krankenhäusern zu einer angstbehafteten Gegenreaktion, selbstbestimmte Kontrolle über die Gestaltung eines würdevollen Todes wiederzuerlangen [17].

Der Kampf der Behandler gegen den Tod, der den Patienten oftmals nur noch als Schauplatz erscheinen lässt, führt unweigerlich zu einer Entmündigung des Sterbenden. Anders als beispielsweise bei einer Zahnextraktion, bei der vielleicht per se nichts gegen die Degradierung des eigenen Körpers zum Objekt ärztlichen Handelns einzuwenden ist, ist der Tod für die eigene Identität von großer Bedeutung. Die Entwertung der Sterbenden zum ausschließlichen Objekt ärztlichen Handelns wird als entwürdigend und entfremdend erlebt [24]. Daher ist es nicht verwunderlich, dass mit dem propagierten Verfassen von Patientenverfügungen ein Höhenflug der Patientenautonomie innerhalb der Medizin einsetzte, auch wenn statistische Erhebungen in Deutschland die Zunahme von Patientenverfügungen nur ungenau belegen [8, 14]. So wie in der Kasuistik stellt eine Patientenverfügung eine Willensfestlegung ungewünschter medizinischer Behandlungsmaßnahmen im Voraus dar, doch bleibt der Anspruch auf Zuwendung und Unterstützung durch die involvierten Akteure aufrecht. Erst die Aspekte der Zuwendung und Unterstützung helfen dabei, ein auf Selbstbestimmung (hier: Patientenverfügung) reduziertes Würdeverständnis zu überwinden und sich umfangreicher, wahrlich würdezentriert dem Patienten zuzuwenden.

So unbestritten wichtig das Abfassen einer Patientenverfügung sein mag, sollte es primär dazu führen, sich bewusst mit Fragen zum Thema würdevoller Behandlung auseinanderzusetzen und sich mit bestimmten Szenarien am Lebensende zu konfrontieren. Vielfach wird jedoch aus den zur Verfügung gestellten Vorlagen von Patientenverfügungen der Eindruck erweckt, dass ein pflegebedürftiges Leben als ungewünscht, ja minderwertig eingestuft wird. Auch hier spielt die mit Abhängigkeit einhergehende schwindende bis verlorene Autonomie, die oftmals zur Einschätzung eines unwürdigen Lebenszustandes führt, eine entscheidende Rolle [17]. Der Patient der Kasuistik scheint zwar seine autonome „Pflicht“ in Form der Patientenverfügung erfüllt zu haben, doch eine gewisse Restunsicherheit bzw. Gestaltungsoffenheit bleibt auch bei einem detailliert im Voraus festgelegten Patientenwillen bestehen und sollte im medizinischen Alltag entsprechenden Raum erfahren.

Bei aller pietätgebotener Privatheit des Todes sollte daraus keine Leugnung jeglichen sozialen Charakters und zwischenmenschlicher Interaktion in dieser Lebensphase resultieren. Die Solidarität droht aus dem Blickfeld zu geraten. Dabei kann die Anteilnahme an Tod und Sterben als soziale Errungenschaft bezeichnet werden [26]. Angewiesenheit als natürlicher Bestandteil von Lebensphasen, die mit Krankheit und Funktionseinschränkung bis hin zum Sterben einhergehen, und als Chance für eine von Beziehung getragene humane Medizin, könnte wieder eine prominentere und positivere Rolle im gesellschaftlichen Diskurs zur *Ars moriendi* erlangen. Palliative Care als wohl einzige darauf spezialisierte medizinische Disziplin wird den Ansprüchen der Versorgung in diesem Lebensabschnitt gerecht, es wäre jedoch angesichts der weit darüber hinausgehenden gesellschaftlichen Relevanz zu kurz gegriffen, dies als hinreichend anzunehmen. Haltungen der Gelassenheit und des Zulassens könnten sowohl in der Medizin als auch sozial als angemessener Umgang mit dem Tod als Bestandteil des Lebens verstanden werden, ohne dabei einer passiven Untätigkeit zu verfallen [17]. Besonders diese Tugenden sind es auch, die einen wesentlichen Beitrag zu einem erweiterten, eigentlich adäquaten Würdeverständnis leisten können.

Fasst man die Forderung nach einem Sterben in Würde reduktionistisch auf, so mag es vielleicht genügen, die Wahrung der Autonomie der Sterbenden und deren Recht auf Zuwendung und Hilfe zu beachten. Es sollte jedoch noch das Moment der Verantwortung miteinbezogen werden, welche sich nicht auf die selbstbestimmte Vorausverfügung des Sterbenden beschränkt [15]. Verantwortung und Autonomie können dabei als dynamische Konzepte unter Einbezug von die Autonomie selbst charakterisierenden Aspekten wie Authentizität und Selbstaktualisierung verstanden werden, im Zuge derer die Patienten die Abhängigkeit von den Helfenden beim Sterbeprozess annehmen. Auch die Verantwortung, die das Umfeld für das Sterben in Würde trägt, ist zu beachten, z. B. durch aufrichtige Zuwendung, das Respektieren der Patientenautonomie und die aktive Auseinandersetzung mit dem Tod. Alle Involvierten, besonders natürlich die Angehörigen der medizinischen und pflegerischen Berufe, sind im Angesicht eines schwer kranken Menschen angehalten, ihr eigenes Menschenbild insbesondere im Kontext von Alter und Tod kritisch zu hinterfragen.

Bei Interesse an einer ausführlicheren Diskussion der Rolle der involvierten Akteure der Kasuistik kann das *Zusatzmaterial online: Supplement 2* aufgerufen werden.

## Fazit für die Praxis

- Im medizinischen Alltag nimmt der Umgang mit Tod und Sterben nach wie vor einen besonderen, aber in Anbetracht einer auf Mortalitätsreduktion ausgerichteten Hochleistungsmedizin an den Rand gedrängten Stellenwert ein. Patientenverfügungen helfen als Ausdruck der Selbstbestimmung, medizinisches Handeln gemäß den eigenen Vorstellungen eines würdevollen Sterbens auszurichten.
- Unerwartete Wendungen im Krankheitsgeschehen, vermeintlich widersprüchliche, aber authentische Verhaltensweisen des Betroffenen oder divergierende Ansichten über den Patientenwillen können zu Verunsicherung und unterschiedlichen Auslegungen der Patientenautonomie führen.
- Basierend auf der Kasuistik werden medizinrechtliche und ethische Besonderheiten beleuchtet, zum anderen zu einem vielschichtigen Konzept der Menschenwürde hingeführt.
- Aufgrund der gegenwärtig ungeklärten deutschen Rechtslage zur Sterbehilfedebatte sollte in vergleichbaren praktischen Szenarien fachkompetenter Rat durch die klinische Ethikberatung oder juristisch eingeholt werden.
- Es wird für eine Rückbesinnung auf tugendhafte Eigenschaften einer angewandten Medizinethik plädiert, in welcher Zuwendung, Selbstentfaltung und Anerkennung der individuellen und medizinischen Grenzen als Chance zur wahren Verwirklichung von Tod und Sterben in Autonomie und Würde dienen.

## Supplementary Information

Supplement 1 Ergänzungen zur Einleitung

Supplement 2 Ergänzungen zur Diskussion

## Footnotes

1 1 Zur besseren Lesbarkeit wird in diesem Artikel das generische Maskulinum verwendet. Die in dieser Arbeit verwendeten Personenbezeichnungen beziehen sich – sofern nicht anders kenntlich gemacht – auf alle Geschlechter.
