# Supplementary material for: Human dignity and autonomy in medicoethical decisions at the end of life
Source: Z Gerontol Geriatr. 2024 May 14;57(7):550–5. [Article in German] doi: 10.1007/s00391-024-02308-1 (PMC11499326; doi:10.1007/s00391-024-02308-1)
Supplement: Supplementary file 1 — Supplement 1 Ergänzungen zur Einleitung [file 391_2024_2308_MOESM1_ESM.docx]

# Supplement 1 Ergänzungen zur Einleitung

## Aspekte der Menschenwürde am Lebensende

Unabhängig vom Lebensende kann man Menschenwürde als „eine Reihe von elementaren moralischen Rechten und Ansprüchen, die dem Individuum allein aus seiner Zugehörigkeit zur menschlichen Gattung erwachsen“ verstehen [1]. Zwei wesentliche Merkmale sind dabei hervorzuheben: Die Würde ist erstens etwas, das dem Menschen allein aufgrund seiner bloßen Existenz, eben weil er Mensch ist, gegeben ist. Zweitens ist Menschenwürde ein objektiver Anspruch, ein Recht, das sowohl von anderen als auch vom Würdeträger selbst Anerkennung und Erfüllung verlangt und nicht von subjektiven Voraussetzungen abhängig ist [3].

Werden nun spezifische Aspekte der Menschenwürde im Kontext von Entscheidungen am Lebensende näher betrachtet, erscheinen zwei ethische Aspekte von besonderer Bedeutung (Vgl. hierzu [3]): Zum einen weisen dem Tode nahestehende Personen eine *Hilfsbedürftigkeit* in vielerlei Hinsicht auf. Jeder Mensch, egal ob unerwartet oder über einen längeren Zeitraum (z. B. im Falle einer langsam progredienten, lebenszeitverkürzenden Grunderkrankung) mit dem nahenden Tod konfrontiert, oder sich ihn herbeisehnend, wird früher oder später Bedarf an medizinischer, pflegerischer, seelsorglicher und/oder psychotherapeutischer Hilfe aufweisen, oder einfach nur um den Beistand einer nahestehenden Person bitten. Und selbst in weitgehender sozialer Isolation kann Hilfsbedürftigkeit im Sinne des Schaffens einer *würdevollen* Atmosphäre zum Ableben bestehen. In der Medizinethik ist das Gebot der Hilfeleistung eines der prominentesten. Anspruch auf und Erfüllung des Hilfsgebotes treffen sich somit an diesem Abschnitt des Lebensendes im Besonderen.

Der zweite besonders berücksichtigungswürdige ethische Aspekt ergibt sich möglicherweise allein aus dem Faktum der *Nähe zum Tod*. Die meisten älteren Menschen werden sich mit Fragestellungen zum Lebensende beschäftigen, je näher sie dem Tode kommen. Viele setzen eine Patientenverfügung auf, bestimmen Angehörige zu Vorsorgebevollmächtigten, suchen vielleicht einfach nur das ernsthafte Gespräch über die Thematik mit anderen oder teilen Nahestehenden – oft auch nur beiläufig – mit, welche Umstände und welchen Umgang sie rund um ihren Tod wünschen bzw. ablehnen; dies alles ja häufig gerade auch mit der Intention, eine individuell als würdevoll verstandene Behandlung sicherzustellen. So könnte man vielen Sterbenskranken ein größeres Wissen um die Transzendenz ihres zeitlich begrenzten Daseins unterstellen, woraus die Annahme resultiert, dem am Lebensende stehenden Menschen eine besondere Form der Würde beizumessen [3].

## Autonomie versus Menschenwürde?

Es gibt prominente soziopolitische Deklarationen wie die Menschenrechtserklärung selbst, die die Menschenwürde als Fundament und Ausgangspunkt heranziehen und Autonomie als essenziellen, aber nicht exklusiven Bestandteil des Gesamtkonzepts Menschenwürde betrachten, und die unmissverständlich den sozialen Charakter beispielsweise des „Geistes der Brüderlichkeit“ erwähnen [9]. Gedanken zu diesem sozialen Aspekt der Menschenwürde sowie zu individuellen Eventualitäten im Kontext der spezifischen Würde am Lebensende seien hier weiter ausgeführt.

Die bereits erwähnten spezifischen Eigenschaften der Würde am Lebensende sind nicht separat von den allgemeinen Aspekten der Menschenwürde zu betrachten. Selbst oder gerade wenn am alternden Menschen bislang unbekannte Verhaltensweisen oder gewisse Persönlichkeitszüge akzentuiert zum Vorschein kommen, die die Würde des Alters zu unterlaufen drohen, so ist in solchen Situationen nie die allgemein zuerkannte Menschenwürde in Frage zu stellen. Freilich stellen solche Erfahrungen Bewährungsproben in der Beziehung mit Angehörigen und anderen Involvierten wie Pflegenden dar; sind es doch meist nicht bekannte, eventuell krankheitsbedingte Züge der Person, die den zwischenmenschlichen Umgang und die Einschätzung der freien Willensbildung so konfliktbehaftet gestalten. Der grundlegende, objektive Anspruch auf Menschenwürde bleibt unabhängig von selbstbestimmter Lebensführung, zeitlebens vollbrachten Errungenschaften oder unliebsamen Umgangsgepflogenheiten bestehen. Zwischen dieser (behaupteten) spezifischen Form der Menschenwürde im Alter und der allgemeinen lassen sich grundlegende diskriminierende Attribute herausarbeiten. Als spezifische Charakteristika seien hier neben dem aus dem Menschenrecht auf Gesundheit abgeleiteten Anspruch auf ein würdevolles Sterben auch die bereits erwähnten, die Nähe zum Tod und die besondere Hilfsbedürftigkeit genannt. Übergeordnet besteht jedoch ein verbindendes Element, denn die allgemeine Würde wird dem Menschen in jeder Lebensphase seiner Entwicklung zuteil (also auch am Lebensende), gleichwohl sie durch würdeloses Verhalten beeinträchtigt werden kann. Kraft der Autonomie kann bereits im Voraus ein wesentlicher Beitrag zum Schutz der individuellen Auffassung von Menschenwürde in Form einer Vorsorgevollmacht oder einer Patientenverfügung geleistet werden.

In konkreten medizinethisch relevanten Konfliktsituationen kann es jedoch zu unterschiedlichen Auffassungen der individuell und fallbezogen zur Debatte stehenden Autonomie bzw. des Patientenwillens kommen, weil beispielsweise unterschiedliche Konfliktparteien die Patientenverfügung divergierend auslegen oder das zuletzt gezeigte und gelebte Verhalten des Betroffenen als nicht authentisch mit der Willenserklärung in der Patientenverfügung übereinstimmend erachtet wird (also Konflikt autonomer versus natürlicher Patientenwille).

Hierbei kann das Konzept der *Authentizität* eine stärkere Gewichtung erfahren und gar in den Rang der Autonomie erhoben werden. Authentizität wird verstanden als eine sowohl von der Person selbst als auch vom Umfeld beurteilte, unverwechselbare und mit den Überzeugungen, Haltungen und Charakterzügen als stimmig erachtete Willensäußerung oder Handlungsweise [2]. Eigentlich wird der Authentizität eine maßgebende Rolle in der Graduierung einer als autonom erachteten Entscheidung zuteil, quasi nach dem Motto: je authentischer, desto autonomer die Entscheidung [10]. Erweist sich eine Willenserklärung oder Verhaltensweise des Kranken als nicht authentisch, gilt es zu hinterfragen und zu analysieren, worauf diese begründet ist. So könnten neben krankheitsbedingten Veränderungen schlichtweg auch eine neu bewertete Lebenseinstellung oder persönlich anders gewichtete moralische Überzeugungen zu einer gewandelten, „neuen“ Authentizität beigetragen haben. Es hat sich ein breites ethisches Diskussionsfeld rund um den Stellenwert der Authentizität im Konzept der Autonomie bzw. bei schwindender Selbstbestimmungsfähigkeit entwickelt, wozu an dieser Stelle nur exemplarisch an entsprechende Literatur verwiesen wird [2, 8, 10].

Mit den Begriffen der Autonomie und Authentizität ist ein eng damit assoziierter zu nennen, nämlich die *Integrität*, welche eine menschliche Fähigkeit zur Akzeptanz des gelebten wie ungelebten Lebens sowie eine als stimmig wahrgenommene Entwicklung und Sinnhaftigkeit des eigenen Lebens beschreibt [5]. Dabei scheint das Entscheidende zu sein, trotz der mit steigendem Alter unvermeidlich einhergehenden und zunehmenden Erfahrung von Verlusten (geliebter Menschen, persönlicher Ziele, Interessen etc.), Vergänglichkeit und Endgültigkeit eine tragfähige Lebensperspektive aufrechtzuerhalten, indem man sich und sein Leben als eine sich stets im Werden befindliche Totalität erfährt [5]. Parallel dazu gibt es eine Debatte in der narrativen Theorie personaler Identität, in der für die Kontinuität personaler Identität ein Mindestmaß an narrativer Kohärenz gefordert wird [7].

Schwinden im Zuge häufig vorkommender Erkrankungen des höheren Lebensalters grundlegende kognitive oder intellektuelle Fähigkeiten des Menschen oder kommen die bereits beschriebenen neuen Verhaltensweisen oder ein (subjektiv) gewandeltes Selbstbild zum Vorschein, so droht, dass das Umfeld die Lebenslage einer Person als weniger menschenwürdig wahrnimmt. Beispielsweise kann eine durch kognitive Funktionseinbußen bedingte verminderte Körperhygiene vom Umfeld als die Würde beeinträchtigend aufgefasst werden, während es vom Betroffenen selbst nicht derart empfunden wird. Oftmals wird dieser Umstand durch körperliche Einbußen, die naturgemäß zu vermehrter Hilfsbedürftigkeit und Abhängigkeit führen, begünstigt. Aus vermeintlich reduzierter bis abhandengekommener Selbstbestimmung oder zunehmender Abhängigkeit wird nur allzu vorschnell auf eine verminderte Menschenwürde geschlossen. Dem stehen jedoch gerade im Konzept eines gelingenden Lebens zwei entscheidende Kategorien in Form *bewusst angenommener Abhängigkeit* und *Selbstaktualisierung* entgegen [4]. Bei der *bewusst angenommenen Abhängigkeit* handelt es sich schlichtweg um ein Merkmal der conditio humana, nämlich die Fähigkeit zur Annahme der eigenen menschlichen Verletzlichkeit in Form von Angewiesensein auf Unterstützung im Falle körperlicher oder geistiger Einschränkungen. Mit ihr können Defizite zumindest teilweise kompensiert und etwaige Folgen verringert werden. Als *Selbstaktualisierung* wird ein qualitativ verschiedenartiges, situativ stimmiges Streben des Menschen nach Verwirklichung von Werten verstanden, d. h. wie ein Mensch seine Werte mit der Art seiner Körperlichkeit, Emotionen, Sinnesempfindungen und sozialen Interaktion lebt [5]. In der Philosophie erfährt diese Ansicht der Bezogenheit zueinander eine besondere Würdigung unter dem Begriff der „relationalen Autonomie“, sowohl in der speziellen Selbstauffassung der Person und ihrer Beziehung(en), in welcher Autonomie zu definieren ist, als auch in der Dynamik der Erwägung und Urteilsbildung [2, 6].

Literatur

1. Birnbacher D (2007) Analytische Einführung in die Ethik. De Gruyter, Berlin, New York

2. Christman J (2004) Relational autonomy, liberal individualism, and the social constitution of selves. Philosophical Studies 117:143–164

3. Härle W (2010) Menschenbild und Menschenwürde am Ende des Lebens. Eine Einführung. In: Fuchs T, Kruse A, Schwarzkopf G (Hrsg) Menschenbild und Menschenwürde am Ende des Lebens. Universitätsverlag Winter, Heidelberg, S 11–26

4. Kruse A (2005) Selbstständigkeit, bewusst angenommene Abhängigkeit, Selbstverantwortung und Mitverantwortung als zentrale Kategorien einer ethischen Betrachtung des Alters. Z Gerontol Geriatr 38:273–287

5. Kruse A (2010) Der Respekt vor der Würde des Menschen am Ende seines Lebens. In: Fuchs T, Kruse A, Schwarzkopf G (Hrsg) Menschenbild und Menschenwürde am Ende des Lebens. Universitätsverlag Winter, Heidelberg, S 27–55

6. Mackenzie C (Hrsg) (2000) Relational autonomy. Feminist perspectives on autonomy, agency, and the social self. Oxford University Press, New York

7. Schechtman M (1996) The constitution of selves. Cornell University Press, Ithaca, NY

8. Sjöstrand M, Juth N (2014) Authenticity and psychiatric disorder: does autonomy of personal preferences matter? Med Health Care Philos 17:115–122

9. http://www.un.org/en/universal-declaration-human-rights/. Zugegriffen: 28. Dezember 2023

10. White L (2018) The Need for Authenticity-Based Autonomy in Medical Ethics. HEC Forum 30:191–209
