# Supplementary material for: Human dignity and autonomy in medicoethical decisions at the end of life
Source: Z Gerontol Geriatr. 2024 May 14;57(7):550–5. [Article in German] doi: 10.1007/s00391-024-02308-1 (PMC11499326; doi:10.1007/s00391-024-02308-1)
Supplement: Supplementary file 2 — Supplement 2 Ergänzungen zur Diskussion [file 391_2024_2308_MOESM2_ESM.docx]

# Supplement 2 Ergänzungen zur Diskussion

Ohne sie als allzu starre Schablone in der Anwendung zu missbrauchen, liefern die erstmals von Tom L. Beauchamp und James F. Childress diskutierten vier medizinethischen Prinzipien, nämlich Autonomie, Schadensvermeidung, Patientenwohl und Gerechtigkeit eine Art Modell, anhand dessen ethische Abwägungen und Gewichtungen zum Entscheidungsprozess beitragen können [1]. Wie bereits einleitend erwähnt, hat in westlichen Kulturen unter anderem nach den historischen Verbrechen der Euthanasie unter dem paternalistischen Deckmantel die Respektierung der Selbstbestimmung (nach Kantischer Tradition) eine gewisse Trumpfstellung unter den verschiedenen Komponenten der Menschenwürde erlangt. Doch zur Achtung der Würde gehören stets auch die Berücksichtigung weiterer Rechte, z. B. auf die Gewährung des Existenzminimums, auf die Freiheit von starken, anhaltenden Schmerzen, auf ein Minimum an (Handlungs-)Freiheit oder Bedingungen der Aufrechterhaltung von Selbstachtung [2]. Diese Aspekte übertreffen ein auf Autonomie reduziertes Konzept der Menschenwürde eindeutig, da beispielsweise die Selbstachtung verletzt werden kann, ohne dabei die Autonomie zu beeinträchtigen.

## Die Menschenwürde des sterbenskranken Patienten

Es ist die situative Gegebenheit, nämlich die Erkrankung bzw. die Konfrontation mit dem Tod selbst, die die besondere Rolle der Würde ausmacht, und nicht ein vorgegebenes abstraktes Konzept. Gerade erst Grenzsituationen dieser Art befähigen, zu einer neuen Lebenseinstellung zu gelangen, ohne dabei vorbestehende persönliche Werthaltungen zu vergessen [3]. Erst die Berücksichtigung aktualisierter Wertvorstellungen und Entscheidungen, wenn auch durch kognitive Einschränkungen beeinträchtigt, liefert einen Beitrag zur Anerkennung und Entfaltung der Patientenwürde [6]. Auch die *Selbstaktualisierung* scheint ein wesentliches Merkmal zu sein, wenn man die teils unerwarteten Handlungs- und Verhaltensweisen des Patienten der Kasuistik versucht zu verstehen. Scheinbar strebt dieser sterbenskranke Mensch nach einer Werteverwirklichung seiner auch bisher gelebten Selbstbestimmung, auch wenn es zunächst nicht dem dargelegten Willen der Patientenverfügung entsprechen mag.

In Folge erschließt sich dann letztlich eine weitere Dimension des Würdebegriffs in Form der *relationalen Würde*, bei der die involvierten Akteure gegenseitigen Austausch und werthafte Aktualisierung pflegen und dabei stets den Patienten, aber auch die Dynamik zwischen Patient und Umfeld im Mittelpunkt behalten [6]. Stoecker schreibt von der sogenannten „bürgerlichen Identität“, an der unsere Würde hängt, worunter er vage und umfangreiche, individuell zu definierende Merkmale unseres Selbst versteht, wie z. B. unsere Persönlichkeit, unsere Biografie und Wesenszüge, aber auch die gesellschaftliche Stellung [8]. Unsere Würde ist bedroht, wenn Umstände am Lebensende diese bürgerliche Identität zu beeinträchtigen drohen.

Würde man auch in diesem Kontext den Würdebegriff reduktionistisch betrachten, das heißt jegliche Dynamik der Persönlichkeitsentwicklung, der Identität, der Aktualisierung von Wertvorstellungen oder der Krankheitsgeschichte außer Acht lassen, dann wäre ein medizinisches Handeln rein an dem auf Autonomie reduzierten Verständnis unzulänglich für eine individuelle würdezentrierte Versorgung.

Die im Supplement 1 erwähnte *bewusst angenommene Abhängigkeit* muss ebenso situations- und kontextbezogen betrachtet werden [4]. Während zu einem früheren Zeitpunkt Abhängigkeit (in Form der künstlichen Ernährung) gemäß Patientenverfügung vom Patienten der Kasuistik als die Würde mit ihrem Teilaspekt der Autonomie verletzend eingestuft worden sein muss, erfährt auch die Einschätzung dieser medizinischen Maßnahme eine Reevaluation: Der Betroffene scheint diese partielle Abhängigkeit bewusst anzunehmen, gleichwohl eine gewisse Autonomie durch die erlernte Selbstbedienung der Magensonde vorhanden ist. Weil es krankheitsbedingt zu einer Beeinträchtigung der zur Selbstbestimmung beitragenden Fähigkeiten wie z. B. Gedächtnisfunktion und Sprachverständnis kommen kann, wird auch die daraus konstituierte freie Willensbildung affiziert [9]. Trotz Einschränkungen solcher Fähigkeiten kann das Individuum noch für rationale Gründe empfänglich sein (Rezeptivität) und seine Entscheidung aufgrund der Abwägung bestimmter Gründe anpassen (Reaktivität; [7]). Auch wenn der natürliche Wille alleine freilich nicht zur Einwilligung in eine Maßnahme ausreicht, kann er einen Beitrag zum Akt der Selbstbestimmung leisten. Meist wird man jedoch mit zunehmendem Schwund neurokognitiver Fähigkeiten (z. B. bei fortgeschrittener Demenz) einem vorab festgelegten autonomen Willen und seiner Verbindlichkeit den Vorrang einräumen [11, 12].

Einmal mehr würde ein rein auf neurokognitive Funktionstüchtigkeit reduziertes Verständnis der freien Willensbestimmung bzw. Autonomie an dem Würdekern der Person und davon beeinflussten medizinischen Handlungs- bzw. Unterlassungsmaßnahmen vorbeigehen. Die Berücksichtigung bzw. kontextsensitive Evaluation, ob die Einschränkung der freien Willensfähigkeit zum gegebenen Zeitpunkt auch zu einer geänderten Beurteilung der individuellen Würde führt, sollte in dem Entscheidungsmoment nicht unberücksichtigt bleiben.

## Die ethische Haltung des Umfelds zur Würde sterbenskranker Menschen

Unter der Voraussetzung einer gegebenen autonomen Willensbestimmung und in Übereinstimmung mit gerade vordergründig relevanten ethischen Überlegungen (z. B. Authentizität) sollte das zum betreffenden Zeitpunkt gezeigte Verhalten des Betroffenen handlungsleitenden Einfluss auf das Behandlungsteam und die Angehörigen haben, um der Patientenwürde gerecht zu werden. Erwächst derivativ ein normativer Anspruch aus der Authentizität, ist jedoch besondere Vorsicht geboten, Authentizität als ausschließliches Übereinstimmungsmodell anzuwenden. Authentisch in diesem Zusammenhang bedeutet, ob Entscheidungen und Handlungen Rückschlüsse auf Haltungen und Einstellungen der Person zum Handlungsziel erlauben. Die Unterscheidung von intentionalen und nicht-intentionalen Handlungen kann dabei Probleme in der Beurteilung von Authentizität bereiten, was gerade bei der Diskussion rund um die krankheitsbedingte Beeinträchtigung des freien vs. des natürlichen Willens besondere Berücksichtigung erfährt (siehe auch Diskussion im Haupttext und [6]).

Welche konkreten Pflichten allen hilfsbedürftigen Menschen, hier der mit dem Tode konfrontierten Person gegenüber bestehen, „ist immer auch abhängig von der konkreten Situation, in der der Mensch sich befindet und in der immer auch die Würde aller anderen an der Situation beteiligten Personen zu achten ist“ [13]. Ärztliches Entscheiden und Handeln erfolgt ebenso situativ. Nichtsdestotrotz dienen ethische Normen als übergeordneter Handlungsrahmen und sollen die Entscheidung oder Handlung im konkreten Einzelfall erleichtern. Die Authentizität sollte daher als ein zwar situativ wandlungsfähiges, aber nicht beliebig formbares Konzept in der Beurteilung von Autonomie dienen. Bei der Abwägung Lebensschutz vs. Würdeschutz muss bei medizinethischen Entscheidungen am Lebensende eine Evaluation nach dem Behandlungs- und Versorgungsziel stattfinden. Es sollte möglichst bereits zum Zeitpunkt der Indikationsstellung geklärt sein, ob die betreffende Maßnahme (hier die der künstlichen Ernährung) dazu geeignet ist, einen präventiven, kurativen oder palliativen Beitrag zu leisten [5]. Diese Einordnung kann aber wie im vorliegenden Fallbeispiel durchaus auch eine andersartige Gewichtung während des Behandlungsverlaufs erfahren. Jedenfalls hilft es auch hier, kein auf Autonomie reduziertes Würdeverständnis zu haben, um dem Behandlungsziel gerecht zu werden.

Bei der Behandlung sterbenskranker Menschen sollte für Pflegende und Ärzte die Konzentration auf das Erleben und Verhalten des Patienten in den Mittelpunkt rücken. Setzt der Kranke sich vermehrt mit dem nahenden Tod auseinander, sollte dies entsprechend durch die Behandler und übrigen Begleiter gewürdigt und adressiert werden. Es muss sich dabei nicht zwangsläufig um einen verbal kommunizierten Akt handeln. Vielmehr können andersartige Interaktionsmuster wie die Möglichkeit des Nicht-Handelns, getragen von einer Haltung der Ruhe und inneren Gelassenheit, eine angemessene und durchaus positiv zu bewertende Antwort auf die bewusste Entscheidung darstellen, sich auf das Sterben vorzubereiten. Dadurch wird nicht Weiterleben um jeden Preis als einziges Ziel vermittelt, sondern eine transformierte Art der Hoffnung durch Loslassen erst ermöglicht [10]. Es geht mit einer Rückbesinnung auf eine ureigentliche Haltung des ärztlichen Ethos einher, nämlich auf den Beistand unheilbar erkrankter Menschen, was abseits der Palliativ- und Allgemeinmedizin kaum mehr als wichtiger qualitativer Bestandteil des Heilberufes erachtet werden mag. Nach wie vor gelten zumeist der quantitativ messbare Therapieerfolg und der (vorübergehende) Triumph über den Tod als Indikatoren erfolgreicher ärztlicher Behandlung. Ein englischer Slogan der Palliativmedizin könnte hier die Sache auf den Punkt bringen: „We can’t always cure but we can always care“. Der allgemeine medizinische Fürsorge- und Heilauftrag soll dadurch selbstverständlich nicht unterminiert werden, der Lebensschutz bleibt oberstes Gebot. Da aber die Würde des Menschen auf der grundlegenden Kondition der jedes Lebewesen ereilenden, zum Tod bestimmten Entwicklung angelegt ist, ist in diesem Lebensschutz auch der Respekt vor dem Sterben begründet: das Lebensende in Freiheit anzunehmen und in Würde erfahren zu dürfen. Das scheint nicht nur dem allgemeinen Würdeverständnis, sondern auch dem im konkreten Fall vorliegenden am nächsten zu kommen.

Gelingt es dem medizinischen Fachpersonal, ein solch erweitertes Würdeverständnis zu verinnerlichen und die beschriebenen Tugenden in der wertschätzenden Kommunikation inklusive Zuhören, im Aushalten von schwindenden kurativen Behandlungsmöglichkeiten und im Verzicht auf voreiligen Aktionismus umzusetzen, wird ein reduktionistisches Würdeverständnis überwunden und dem individuellen Würdekonzept gerecht.

## Fazit zum Fallbeispiel

Wäre man bei dem Patienten in der Kasuistik strikt seiner Selbstbestimmung gemäß Patientenverfügung gefolgt, hätte es keine PEG-Sondenanlage und ergo auch keine Bauchdeckenentzündung mit letalem Ausgang gegeben. Vielleicht hätte der Patient dann noch einige Wochen oder Monate länger zu leben gehabt. In der Beachtung eines vielgestaltigen Konzepts der Menschenwürde mit individuellen Besonderheiten resultierte jedoch auch ein umfangreicheres Verständnis der Autonomie und man konnte den Patientenwünschen schließlich Rechnung tragen, auch wenn divergierende Ansichten seitens der Angehörigen bzw. Behandler bestanden. Die ethische Entscheidungsfindung in dem Fall war jedenfalls argumentativ suffizient begründet und der theoretische potenzielle Gewinn an Lebenszeit im Falle der unterlassenen PEG-Sondenanlage wäre womöglich weniger im Sinne des Patienten gewesen als die bis zum Versterben gewährte Lebensqualität. Es lässt sich festhalten, dass gerade in dieser Kasuistik der besonders berücksichtigungswürdige Schutz vulnerabler Personengruppen im Kontext von Entscheidungen am Lebensende gemäß UN-BRK zum Vorschein kommt, schließlich aber dem aus der Menschenwürde erwachsenen differenzierten Konzept der Selbstbestimmung Rechnung getragen wurde.

Literatur

1. Beauchamp TL, Childress JF (2019) Principles of biomedical ethics. Oxford University Press, New York

2. Birnbacher D (1996) Ambiguities in the Concept of Menschenwürde. In: Bayertz K (Hrsg) Sanctity of Life and Human Dignity. Springer, Dordrecht, S 107–121

3. Frankl VE (2016) Der Wille zum Sinn. Hogrefe, Bern

4. Kruse A (2005) Selbstständigkeit, bewusst angenommene Abhängigkeit, Selbstverantwortung und Mitverantwortung als zentrale Kategorien einer ethischen Betrachtung des Alters. Z Gerontol Geriatr 38:273–287

5. Lorenzl S (2010) Flüssigkeit und Ernährung am Lebensende. Entscheidungsfindung und medizinisch-ethische Problembereiche. Z Med Ethik 56:121–130

6. Paul NW (2013) Ethische Dimensionen der künstlichen Ernährung bei Intensivpatienten. In: Rümelin A, Mayer K (Hrsg) Ernährung des Intensivpatienten. Springer, Berlin, S 119–128

7. Schulte P (2010) "Willensfreiheit". In: Sandkühler HJ (Hrsg) Enzyklopädie Philosophie. Felix Meiner Verlag, Hamburg, S 2997–3003

8. Stoecker R (2019) Theorie und Praxis der Menschenwürde. mentis, Paderborn

9. Thiele F (2011) Autonomie und Einwilligung in der Medizin. Eine moralphilosophische Rekonstruktion. mentis, Paderborn

10. Verres R (1998) Vom Handlungsdruck zur inneren Ruhe. In: Verres R, Klusmann D (Hrsg) Strahlentherapie im Erleben der Patienten. Barth, Heidelberg, S 111–116

11. Vollmann J (2001) Advance directives in patients with Alzheimer's disease. Ethical and clinical considerations. Med Health Care Philos 4:161–167

12. Walensi M, Inthorn J, Paul NW (2016) Willensfreiheit, Determinismus und die Abwägung eines vorab erklärten autonomen Willens im Falle einer natürlichen Willensäußerung. Int Z Philos Psychosom 1:1–16

13. https://www.bpb.de/gesellschaft/umwelt/bioethik/33733/wuerde-argumente. Zugegriffen: 06. Oktober 2023
